# Appendicitis: Oschner’s and Deaver’s Methods Compared*Read at June meeting; Austin District Medical Association.

**Published:** 1903-07

**Authors:** W. J. Matthews

**Affiliations:** Austin, Texas


### TEXAS MEDICAL JOURNAL.

ESTABLISHED JULY, 1885.

PUBLISHED MONTHLY.—SUBSCRIPTION $1.00 A YEAR.

VOL. XIX. AUSTIN, JULY, 1903. No. 1.

### Original Contributions.

For Texas Medical Journal.

### Appendicitis: Oschner's and Deaver's Methods Compared.*

BY W. J. MATTHEWS, M. D., AUSTIN, TEXAS.

When we remember the frequency of this affection, the rapidity with which the disease may proceed from bad to worse, the fatality in a large percentage of cases, its marked tendency to recur in those who are apparently recovered from an attack, we may well consider appendicitis the most important intra-abdominal lesion of today.

The operation for appendicitis is a difficult one, and requires considerable anatomical knowledge and surgical experience on the part of the operator. Such anatomical knowledge and operative skill is rare among those whom we might term occasional operators—a term which would apply to a great number of us. One of the most experienced men in this country in the surgical treatment of appendicitis makes the following statement: "There are thousands of surgeons who are otherwise competent—competent to perform the ordinary surgical and gynecological operations—whom I would not think of permitting to open my abdomen in case I personally suffered from an attack of appendicitis." Professor Oschner, of Chicago, an eminent surgeon whose views this paper reflects, says that he endorses every word of the above utterance. If, then, any treatment is available which should tide a case of appendicitis through the attack safely until a competent operator could be found, or until a more favorable time for operation might arrive, such treatment would surely be hailed as a God-send by every conscientious physician. The treatment emphasized in this paper is that of Pro-

*Read at June meeting; Austin District Medical Association.

fessor Oschner, of Chicago, who has had a vast experience in the treatment of appendicitis. He would always insist on operation at the proper time, when a skilled operator could be found, but says most emphatically that if a competent surgeon is not available that the patient's chances of recovery are many times greater with proper non-surgical treatment than with an operation. In order to make clear the treatment set forth in this paper, let us first consider the anatomical position of the appendix.

The appendix is so situated that it is easily isolated from the general abdominal cavity, if the surrounding structures can be placed in a state of rest. Under a condition of rest the most important physiological function of the cæcum, the omentum and the small intestines consists in surrounding any diseased organ. If the appendix is in a state of inflammation, be it catarrhal, septic or gangrenous, the surrounding structures will at once apply themselves around this organ, so as to wall it up from the general peritoneal cavity, thus effectually preventing the escape of septic matter into this cavity. It is a well-known fact that it is the physiological business of the omentum to protect the various intra-abdominal organs against infection in case of danger. The small intestines likewise apply themselves to the omentum, re-enforcing its efforts. The appendix, thus protected, can not infect the general peritoneal cavity. It does not matter whether the patient suffers from catarrhal appendicitis or whether the appendix is gangrenous or perforated, if proper rest of the appendix and surrounding structures is obtained, the patient will invariably recover, and the worst that can happen will be a circumscribed abscess, which the merest tyro in surgery can open, if he only has the sense to stop there.

Next, how is this rest of appendix and surrounding structures obtained? First of all, wash out the stomach with a stomach tube, and keep irrigating until every vestige of undigested food has been washed out of the stomach. It is a well-known fact that while the stomach is being washed out the contents of the small intestines regurgitate into the stomach, so that practically the stomach and small intestines are emptied of all fermenting, undigested matter. It is also well known that as soon as food has passed the pyloris, peristaltic action at once commences in the small intestines, and, instead of assisting the omentum in preventing infection of the peritoneal cavity, this motion will tend to distribute any septic material with which the intestines may have come in contact. Besides, no matter what quantity of food is taken into the stomach, it will always produce gas, which must either be expelled through the œsophagus by eructation or it must pass into the colon through the ilio-cæcal valve, which invariably disturbs the lower end of colon, and this in turn causes serious disturbance in the inflamed appendix. Besides emptying the stomach and small intestines by gastric lavage, withhold all manner of food after irrigation. The patient should absolutely swallow no liquid food, not even beef peptonoids, from one to three weeks, if necessary. Large rectal injections are considered dangerous. If given at all, they should be small, as they are apt to set up peristaltic action. Deaver, in his work on "Appendicitis," relates a case where a large injection was thrown right into the peritoneal cavity, which he afterwards verified by an operation. That this can happen, I am quite sure. I had a case of appendicitis in which a fistulous opening in the cæcum remained for quite a while after the operation. One day I gave him a fairly large enema and soon water welled out of the fistulous track in groin. I repeated this several times with same result. It also occurred in a patient at the City Hospital under similar circumstances. Pardon this digression, I only desired to emphasize the danger of large enemas in appendicitis. Then it will be evident to all that in the non-operative treatment advocated in this paper, we first obtain absolute rest for the diseased organ—just the course we would pursue in inflammation of any other part of the body—and, secondly, we isolate the diseased organ by surrounding it with omentum and small intestines for the purpose of favoring resolution of the inflammatory condition, and if this is not possible, substituting a comparatively harmless circumscribed for a very dangerous diffuse peritonitis. The strength of the patient must, of course, be sustained by nutritive enemata. Professor Oschner prefers an enema consisting of liquid beef peptin—one ounce—and normal saline solution—four ounces—to be repeated every four or six hours.

I have treated two cases strictly following Professor Oschner's views, and the results have simply amazed me. In neither case did I give any opiates. A few hours after washing out the stomach the temperature fell two or three degrees. It was also most remarkable how soon the severe pain disappeared. Nor is this to be wondered at, because I believe that the spasmodic action of the appendix which causes pain is started by the irritation due to the passage, or the attempt at passage, of gas through the ilio-cæcal valve. But when the stomach has been thoroughly washed out and all food is absolutely prohibited afterwards there is nothing in stomach or intestines to ferment, and if there is no fermentation, there can be no peristaltic action of bowels and nothing to make pain. This treatment is certainly life-saving. For example, there are not a few cases in which an operation is absolutely refused, and I know of no treatment that would compare with this in saving life under such circumstances. Again, when a competent operator can not be found this treatment should receive a fair trial. There are also other cases in which an expert surgeon would not dare to operate, on account of the desperate condition of the patient. To such cases Oschner refers. He states that the patient with a temperature of 103, pulse 140, considerable meteorism, costal respiration, intense tenderness over the whole abdomen, with rigidity of the muscles and symptoms of collapse, will die almost invariably without even recovering from the shock of the operation. Place this same patient upon exclusive rectal alimentation after emptying the stomach of its load of septic decomposing material by gastric lavage, and the patient's pain will disappear within twelve hours, the temperature will fall from 1 to 3 degrees in twenty-four hours, the symptoms of collapse will leave, and the tenderness and meteorism will have decreased. A circumscribed abscess may form, which will require opening, or there may be absorption of all the products of inflammation, and when the abdomen is opened a month later nothing may be left but diffuse adhesions. If the abdomen is opened a year later, even these adhesions may have disappeared to a great extent. Furthermore, this treatment will be of immense value to those physicians who never operate at all. They can by this treatment bring their appendicitis cases safely through the attack with credit to themselves and safety to their patients.

I am aware that these views are not accepted by many of the best surgeons of the United States. The battle is still on between the radical school of surgeons, which demands immediate operation in every case of appendicitis, and the conservative school, which advises the interval operation where possible after tiding the patient over an acute attack. On one thing the members of both schools are in the main agreed. The patient should be operated upon at once if seen at the beginning of the attack, namely, the first twenty-four or thirty-six hours; but after that period they differ greatly. The first fears the presence of pus in the abdomen and demands its immediate removal; the second insists that operation at that period before the parts are properly walled off by nature places the patient in danger of peritoneal infection. Two leading members of these schools are Dr. John B. Deaver, of Philadelphia, and Dr. A. J. Oschner, of Chicago, whose views I reflect in this paper.

The latest utterances of Dr. Deaver are as follows: We are satisfied that in years gone by many valuable lives have been sacrificed by waiting for an attack of appendicitis to subside in order to perform the operation of election between attacks. That terrible uncertainty which always exists in appendicitis no longer should exist, for we are aware that if an attack is permitted to advance to the pus-formation stage that we may have any or all of the complications and sequelæ. We are aware that some cases of appendicitis will temporarily improve, but if we follow these long enough there is an entirely different tale unfolded. To the statement that an amelioration of symptoms in acute appendicitis should always be regarded as favorable and a contra-indication to operation, we take exception. The progressing pathologic process is so uncertain and so difficult, yes, impossible, to decipher by the means we possess, exclusive of opening the abdomen, that we clinch this opportunity to operate and thus save lives that otherwise would be lost in waiting for convalescence and the interval stage, which too often does not arrive. We are sure that many will take exception to this; but, nevertheless, we are forced from our experience to decry such exceptions as false. Even though the symptoms apparently ameliorate, the pathologic process is too frequently hurrying on to the stage of pus formation. To follow this teaching is to practice the rule that proves the best in the majority of instances. The oft-repeated old, old story of procrastination is nowhere better illustrated than in connection with the delay for the interval operation. There is a temporary improvement under the application of the ice-bag and administration of laxatives, which, however, should not mislead anyone, as it often is immediately followed by an exacerbation of the symptoms of the disease indicating a perforation of the appendix or the formation of pus or its dissemination. Even while the apparent lull in the appendical storm is on grave complications are making headway in the abdomen, out of sight and out of reach of the examining finger, and constitutional evidences of the disease are not made manifest until irreparable damage has been done and too often a dear and valuable life sacrificed.

Dr. Oschner, of Chicago, also believes in immediate operation if this can be performed during the first twenty-four or thirty-six hours of the attack. He believes that during this time all of the infectious material is still within the walls of the appendix and can consequently be removed without coming in contact with any other portion of the peritoneal cavity, and for the following reasons under these conditions the patient will almost always recover. The appendix can be removed with comparative ease.

There is no occasion for draining the abdominal cavity, consequently no likelihood of the occurrence of a ventral hernia following the operation. Besides, an early operation prevents extensive adhesions, which might impair the future health of the patient. So far most surgeons are agreed, but from this point on they differ. Dr. Oschner, after the attack has lasted forty-eight hours, favors waiting until all the acute symptoms have disappeared for at least two weeks and to remove the appendix at that time, because then, again, he says, all of the advantages which he has mentioned in connection with the operation during the very beginning of an acute attack obtain in every particular. Dr. Oschner in former years used to operate on what he terms desperate cases, to which he was called one week after the attack, and almost in every instance lost his patient; but under his late system of rectal alimentation and stomach washing he is able by operation to save the lives even of these desperate cases.

Now for the statistics as to the results of the operation for appendicitis by these eminent surgeons Drs. Deaver and Oschner. I am glad to be able to give you the most recent statistics.

Each operated on 416 cases. Of Dr. Deaver's 416 cases, 275 were acute and 141 were chronic. Of Dr. Oschner's 416 cases, 263 were acute and 153 were chronic, which shows a striking similarity between the two set of cases. Dr. Deaver's mortality in the acute cases was 37 deaths, and 1 in the chronic. In Dr. Oschner's 263 acute cases there were 10 deaths, and in the chronic cases 2 deaths. Dr. Oschner's mortality is less than 4 per cent. in the acute cases, while Dr. Deaver's is more than 13 per cent. With such figures before us, surely we should give Dr. Oschner's method a fair and impartial trial.

